# Network analysis applied to post-concussion symptoms in two mild traumatic brain injury samples

**DOI:** 10.3389/fneur.2023.1226367

**Published:** 2023-07-20

**Authors:** Josh W. Faulkner, Alice Theadom, Deborah L. Snell, Matt N. Williams

**Affiliations:** ^1^Te Herenga Waka – Victoria University of Wellington, Wellington, New Zealand; ^2^TBI Network, Auckland University of Technology, Auckland, New Zealand; ^3^University of Otago, Dunedin, New Zealand; ^4^Massey University, Auckland, New Zealand

**Keywords:** mild traumatic brain injury, post-concussion symptoms, network analysis, outcomes, concussion

## Abstract

**Objective:**

A latent disease explanation cannot exclusively explain post-concussion symptoms after mild traumatic brain injury (mTBI). Network analysis offers an alternative form of explanation for relationships between symptoms. The study aimed to apply network analysis to post-concussion symptoms in two different mTBI cohorts; an acute treatment-seeking sample and a sample 10 years post-mTBI.

**Method:**

The treatment-seeking sample (*n* = 258) were on average 6 weeks post-injury; the 10 year post mTBI sample (*n* = 193) was derived from a population-based incidence and outcomes study (BIONIC). Network analysis was completed on post-concussion symptoms measured using the Rivermead Post-Concussion Questionnaire.

**Results:**

In the treatment-seeking sample, frustration, blurred vision, and concentration difficulties were central to the network. These symptoms remained central in the 10 year post mTBI sample. A Network Comparison Test revealed evidence of a difference in network structure across the two samples (*p* = 0.045). However, the only symptoms that showed significant differences in strength centrality across samples were irritability and restlessness.

**Conclusion:**

The current findings suggest that frustration, blurred vision and concentration difficulties may have an influential role in the experience and maintenance of post-concussion symptoms. The impact of these symptoms may remain stable over time. Targeting and prioritising the management of these symptoms may be beneficial for mTBI rehabilitation.

## Introduction

There is a growing shift in recognising the frequency, persistence, and impact of post-concussion symptoms following mild traumatic brain injury (mTBI). The previously held belief that mTBI is predominately an acute event, with only a small minority of individuals experiencing persisting symptoms, is no longer supported (1–4). A substantial proportion of people can experience post-concussion symptoms that can persist for several months and sometimes even years (4–7). The implications of persistent post-concussion symptoms can be profound resulting in high disability, lower quality of life and increased use of healthcare resources (8–10). Thus, a better understanding of the aetiology of these symptoms is needed to inform intervention and prevention.

Historically, the term “post-concussion syndrome” has been used to conceptualise and explain the persistence of post-concussion symptoms after mTBI (11). However, the use of this term has been contentious (12). ‘Syndrome’ implies that the resultant symptoms are explained by a single disease entity, in this case, mTBI; yet this explanation has not been supported empirically (3, 13, 14). One problem is that post-concussion symptoms do not always cluster in the same and predictable manner (15, 16). This is not consistent with the symptoms truly representing a specific, cohesive, and predictable syndrome. In addition, post-concussion symptoms tend to be positively correlated (i.e., people who have worse headaches tend also to be more likely to experience concentration impairments, and blurred vision, etc.) (17). The severity of mTBI also does not appear to be a consistently strong predictor of post-concussion symptoms (18). Several studies have found a limited association between mTBI severity indices (such as loss of consciousness and post-traumatic amnesia) and acute or chronic post-concussion symptoms (19–21). In addition, research comparing the severity of post-concussion symptoms and functional outcomes based on the presence of acute intracranial abnormalities has been remarkably mixed (22). Although the term post-concussion symptoms might suggest otherwise, these symptoms are also not specific to TBI and frequently occur following trauma (23, 24), as well as in healthy adults and children (9, 25).

Finally, it is generally agreed that many factors contribute to the development and maintenance of post-concussion symptoms (3, 14). This includes the biological effects of the TBI, psychological and psychosocial factors, chronic pain, pre-injury vulnerabilities, demographic factors and personality characteristics (26–30). However, none of these factors has emerged as a latent common cause (3, 31). Instead, current conceptualisations assert that post-concussion symptoms are multifactorial in causation in accordance with a biopsychosocial framework (14, 18, 28, 32). Thus, a latent model or common cause theory for post-concussion symptoms is inconsistent with current understandings of the aetiology of post-concussion symptoms and prevailing biopsychosocial conceptualisations.

Recently, a new psychometric approach has become popular in psychology that provides an alternative to *latent variable* (common factor) explanations for symptom covariance. This is the *network* perspective (33). In network theory, symptoms can be conceptualised as *nodes* which can be connected by *edges*. Edges can be directed (representing directional causal effects) or undirected (where the direction of causality, if any, is unknown). The network perspective provides a new form of explanation for relationships observed between sets of symptoms: Rather than just being caused by an underlying disease entity, specific symptoms may have causal effects *on one another* (33, 34). There have been calls to apply network analysis to post-concussion symptoms (31). This approach would suggest that post-concussion symptoms co-occur because they are strongly interrelated, activating, amplifying, and mutually reinforcing, not because they arise from a common latent disease entity (31). A network approach may provide an explanation as to why a set of variables are correlated with one another. Thus, adopting a network perspective could lead to new insights into our understanding of the development and maintenance of post-concussion symptoms. This approach makes it possible to examine the architecture of post-concussion symptoms and identify symptoms that are more central and strongly interconnected (31). This has the potential to provide initial targets for treatment and rehabilitation. That is, focusing on one or two symptoms that have a high degree of centrality within the network may dampen or even ameliorate other post-concussion symptoms. This has the potential to result in more effective and less labour-intensive treatment.

Two recent studies have used networks to estimate relationships among common post-concussion symptoms in uninjured adolescents at baseline (before commencing the sports season) (35, 36). These studies found that feeling more emotional and dizziness were central baseline symptoms in adolescents with a history of mental health difficulties (35) and in adolescents with attention deficit hyperactivity disorder (36). Recently, Preszler et al. (37) applied network analysis to post-concussion symptoms in 326 adolescents recruited from a concussion speciality clinic (≤28 days post-injury); post-concussion symptoms were assessed using the Post-Concussion Symptom Scale (PCSS). Dizziness and sadness were the most central symptoms (37). A further study, used this approach, in high school athletes with suspected sports-related concussion also using the PCSS. Difficulty concentrating was the most central and influential symptom in the network (38).

In summary, there appears to be value in applying network analysis to understand the relationships between symptoms that are commonly evident after mTBI. However, existing published studies have produced divergent networks in relatively narrow populations limiting their generalisability. To the best of our knowledge, there is limited research applying this approach to civilian, community-based adult mTBI samples. Although a network analysis could be of value to various aspects of mTBI recovery, understanding the centrality of symptoms within a network of post-concussion symptoms in individuals seeking treatment could help clinicians target treatment efforts to mitigate the development of persistent symptoms. In addition, applying network analysis to individuals with historical mTBI (i.e., 10 years ago) could provide novel insights into the interrelationships among these symptoms over time. These are the overall objectives of the current study. Specifically, this study aimed to (i) estimate networks of post-concussion symptoms in a sample of adult participants seeking treatment for mTBI early after injury and a sample of participants who experienced mTBI 10 years prior, (ii) determine which symptoms are most central (i.e., strongly connected to other symptoms) in each sample network to identify key areas for intervention, and (iii) explore differences in symptom network between in the two samples to examine change in post-concussion symptom networks over time.

## Materials and methods

**Treatment seeking mTBI**: this sample consisted of data collected as part of two studies using similar prospective observational methods. Participants were recruited from outpatient clinics providing rehabilitation services for mTBI across both the North and South Islands of New Zealand between February 2019 and October 2021, obtained independently across two different studies with similar recruitment methods and inclusion criteria (39, 40). All participating clinics were funded by New Zealand’s government-funded injury insurance scheme. At recruitment sites, eligible participants (*n* = 337) were approached by a clinician from the outpatient clinic within 3 months of entry into the service and invited to participate. Eligibility criteria for participants were: (1) aged 16 years or older, (2) sustained an mTBI according to World Health Organization Neurotrauma Taskforce criteria (19), (3) were less than three months post-injury at enrolment, (4) were fluent in English, and (5) had no prior neurological condition or severe unstable medical condition, including a past history of severe traumatic brain injury. Eligible and consenting participants completed questionnaires via REDCap (41), a secure web-based platform (*n* = 252), by mailed questionnaires (*n* = 5), or by telephone (*n* = 1). 64 participants did not complete the assessment and 15 withdrew from the study. Ethical approvals were received from New Zealand’s National Health and Disability Ethics Committee (ref 18/CEN/79) and the Auckland University of Technology Ethics Committee (ref 20/32).

**10 year post-mTBI**: this sample came from a population-based incidence and outcomes study (BIONIC (42)) which registered all cases of TBI that occurred in Hamilton city (urban) and the Waikato District (rural) region of New Zealand between 01/03/10 and 28/02/2011. Participants were recruited through a wide range of strategies including schools and sports clubs, GPs, allied health professionals as well as from the hospital. The original study included people of all ages and TBI severities (*n* = 1,369). All people eligible for the study were invited to complete a series of assessments at baseline (within 2 weeks of injury), one month, six months, one, four, eight, and 10 years after their injury. For the purposes of this analysis, data for adults (who were 16 years or older at the time of the 10 year follow-up assessment) who experienced an mTBI and who completed the Rivermead Post Concussion Symptoms Questionnaire ten years after their injury were included in this analysis. At 10 years following injury eligible participants (*n* = 404) who indicated an interest in further research were contacted via each participant’s preferred mode of contact (e.g., phone, text, mail, email or via social media). Participants were sent a participant information sheet/consent form and were asked to contact the research team if they wanted to participate. Following an expression of interest, participants were sent a link to an online questionnaire using the REDCap database. 140 participants were not able to be contacted, 47 did not complete the study measures, 13 declined to participate and 4 did not participate for unknown reasons.

### Measures

For the mTBI treatment-seeking sample and the 10 year-post mTBI sample a range of measures were administered. In this study, the relevant measure was the 16-item Rivermead Post Concussion Symptom Questionnaire (43) (Cronbach’s alpha = 0.90). Each item specified a symptom (e.g., “headaches,” “nausea and/or vomiting”), and participants were asked to rate their experience in the previous 24 h. These response options were 0 = not experienced at all; 1 = no more of a problem than before injury; 2 = a mild problem; 3 = a moderate problem; 4 = a severe problem. In line with recommended practice “No more of a problem” was rescored as 0 rather than 1 (43).

### Data analysis

In the mTBI treatment-seeking sample 0.4% of data points were missing among participants and 1% of data points were missing in the 10 year post-mTBI sample. Parameter selection in networks was conducted using the EBICglasso algorithm, which selects edges using a graphical lasso (34). Pairwise complete observations were used when calculating the input correlation matrix for the EBICglasso algorithm. The tuning parameter for the lasso is chosen using the Extended Bayesian Information Criterion, which in turn has a tuning parameter *gamma* (which we left at its default of 0.5).

Network parameters were estimated via full information maximum likelihood using the psychonetrics (44) and qgraph packages (45). The only variables in the network models were the 16 symptoms in the Rivermead Post Concussion Symptom Questionnaire. Full information maximum likelihood permits the inclusion of cases and variables with missing data but has the limitation of assuming the variables have a multivariate normal distribution. Taken literally, this assumption is breached because the original data stems from ordered-categorical items (whereas the multivariate normal distribution is continuous). We also completed a version of our analyses using diagonally weighted least squares estimation, which does not permit missing data but does not assume multivariate normality and is thus more robust to the use of ordered-categorical data. The results for these analyses were similar to those presented here and can be found in the Supplementary material. As is the case for any statistical method, network analyses can be vulnerable to sampling error and imperfect replicability. Some recent findings suggest that networks can be *especially* vulnerable to poor replicability (46). One heuristic for assessing the replicability of networks is the “case drop” bootstrap method, where a subset of cases is randomly dropped from a sample, edge weights and centrality are recalculated, and then correlated with the edge weight and centrality estimates from the full sample. If the findings are relatively stable, dropping a small proportion of cases should result in edge and centrality estimates in the bootstrap sample that remain strongly correlated with those in the full sample. Such bootstrapping can be conducted repeatedly, with differing proportions of cases excluded. We adopted this approach to assess the stability of network edges and centrality by using the case-drop strategy in the bootnet package (34). The Fruchterman-Reingold algorithm was used for network plotting (47), resulting in a plot where the distance between nodes corresponds approximately to the strength of their connections. All analyses were conducted in R (R Core Team), version 4.2.1 (48). Skewness and kurtosis statistics were calculated using the moments package (49).

## Results

A summary of the demographic characteristics of the treatment-seeking mTBI and 10 year post-mTBI samples (at 10 year follow-up) is presented in Table 1.

**Table 1:** Demographic characteristics of participants.

	Treatment Seeking mTBI (*n* = 258)	10 year post mTBI (*n* = 193)
Age [M (SD, range)]	36.90 (14.2, 17.0–76.0)	39.5 (17.3, 16.0–86.0)
Sex [*N* (%)]		
Female	164 (63.6)	85 (44.3)
Male	94 (36.4)	107 (55.7)
Ethnicity category [*N* (%)]		
NZ European	179 (69.4)	142 (74.0)
Māori or Pasifika	26 (10.1)	44 (22.9)
Other	53 (20.5)	6 (3.1)
Education History		
Secondary school or less	93 (36.0)	58 (30.0%)
Post-secondary school qualification	165 (64.0)	105 (54.4%)
Unknown		30 (15.6%)
Employment status [*N* (%)]		
Employed	175 (67.8)	124 (61.4)
Not in Employment	83 (32.3)	69 (38.6)
Time Since Injury [M, range]	6.31 (2.00–14.00) weeks	10.0 years (9.25–10.25 years)
Mechanism of Injury [*N* (%)]		
Motor Vehicle Accident	37 (14.3)	40 (20.9)
Hit by object	91 (35.3)	46 (23.8)
Fall	93 (36.0)	66 (34.2)
Assault	23 (9.3)	34 (17.6)
Other	14 (5.5)	7 (3.7)

### Descriptive statistics

Descriptive statistics for the RPQ for each sample are displayed in Table 2. Symptoms were positively correlated across participants, with a median correlation between symptom pairs of 0.40 (min = 0.11, max = 0.75) in the mTBI treatment-seeking group and 0.37 (min = 0.10, max = 0.70) in the 10 year post mTBI group.

**Table 2:** Descriptive statistics for RPQ items.

Item	mTBI Treatment seeking				10 year post mTBI				
	Abbrev	M	SD	Skew	Excess kurtosis	M	SD	Skew	Excess kurtosis
Headaches	hdache	2.38	1.3	−0.75	−0.49	1.58	1.35	0.00	−1.38
Feelings of dizziness	dizzy	1.72	1.4	−0.09	−1.38	1.16	1.27	0.47	−1.15
Nausea and/or vomiting	nausea	1.01	1.32	0.74	−1.05	0.64	1.12	1.46	0.90
Noise sensitivity (easily upset by loud noise)	noise	2.02	1.38	−0.34	−1.11	0.93	1.29	0.89	−0.73
Sleep disturbance	sleep	2.03	1.45	−0.26	−1.23	1.69	1.42	−0.02	−1.39
Fatigue, tiring more easily	fatigue	2.84	1.19	−1.13	0.65	1.64	1.41	0.05	−1.34
Being irritable, easily angered	irrata	2.02	1.41	−0.36	−1.21	1.40	1.31	0.12	−1.45
Feeling depressed or tearful	depress	1.6	1.49	0.16	−1.43	1.22	1.29	0.35	−1.39
Feeling frustrated or impatient	frustrat	2.04	1.37	−0.33	−1.04	1.31	1.28	0.18	−1.47
Forgetfulness, poor memory	forget	2.19	1.33	−0.48	−0.81	1.68	1.32	−0.10	−1.24
Poor concentration	concent	2.36	1.25	−0.74	−0.35	1.23	1.26	0.32	−1.28
Taking longer to think	long_th	2.38	1.23	−0.72	−0.25	1.33	1.29	0.25	−1.29
Blurred vision	blur_vis	1.11	1.38	0.66	−1.11	0.62	1.13	1.54	1.06
Light sensitivity	light_s	1.67	1.51	0.08	−1.5	0.80	1.28	1.23	0.06
Double vision	2x_vis	0.5	1.06	1.81	1.79	0.32	0.90	2.69	5.95
Restlessness	restle	1.28	1.35	0.36	−1.37	1.87	1.28	−0.36	−1.07

### Network analysis

#### mTBI treatment seeking sample

**Figure 1:**
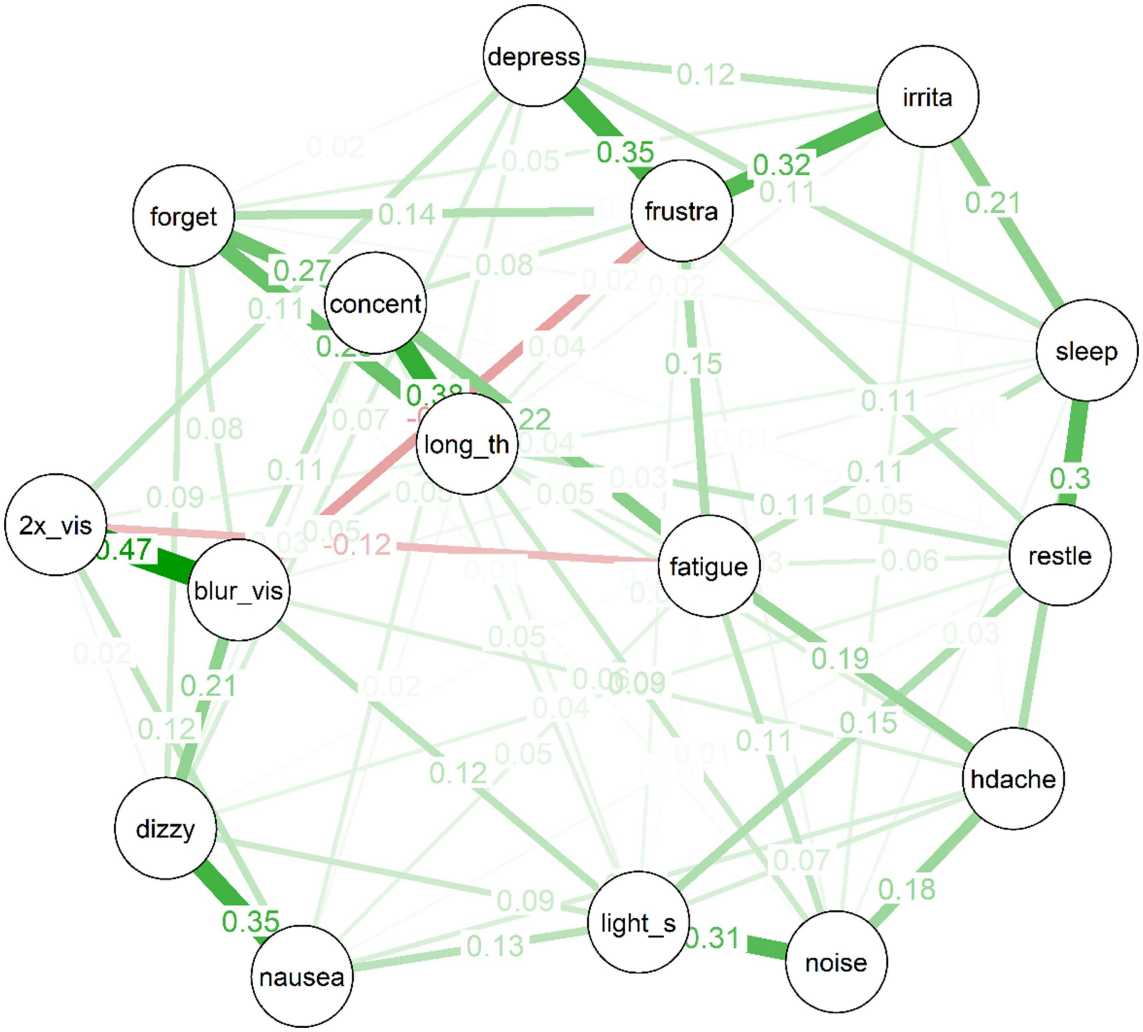
Partial correlation network of the treatment-seeking sample.

The estimated network for the treatment-seeking sample is displayed in Figure 1. In the figure, thicker edges represent stronger connections. Importantly, the edges in this network are *partial* correlations, which means that they represent relationships between pairs of items while controlling all remaining nodes in the network. This means that they more plausibly represent causal effects between symptom pairs than would zero-order correlations (although the directions of any effects remain unknown, and it remains possible that third variables outside the network could produce spurious relationships). Because the edges have been subject to a regularisation process via LASSO during which some edges were removed, there is evidence in favour of the existence of each included edge in the population (even the relatively small edges).

Several interesting features are apparent in the network for the treatment-seeking group. First, items cluster to some extent into “communities”: For example, the mood symptoms of depressed mood, frustration and irritability are clearly connected. So too are the cognitive symptoms of forgetfulness, poor concentration, and “taking longer to think.” Visual disturbances (i.e., blurred vision and double vision) also clustered strongly together, alongside nausea and dizziness; and there was a cluster of more general physical symptoms (i.e., headache, light and noise sensitivity).

Second, almost all of the edges are positive. The strongest edges in the network included are those between blurred vision and double vision, dizziness and nausea, concentration difficulties and taking longer to think, frustration and depression, frustration and irritability, noise sensitivity and light sensitivity, and sleep disturbances and restlessness. Third, the network is of moderate density: Of a possible (16*15)/2 = 120 edges between items, the EBICglasso selected 75 edges, while the remaining 45 were constrained to zero, and are not displayed in the network plot.

**Figure 2:**
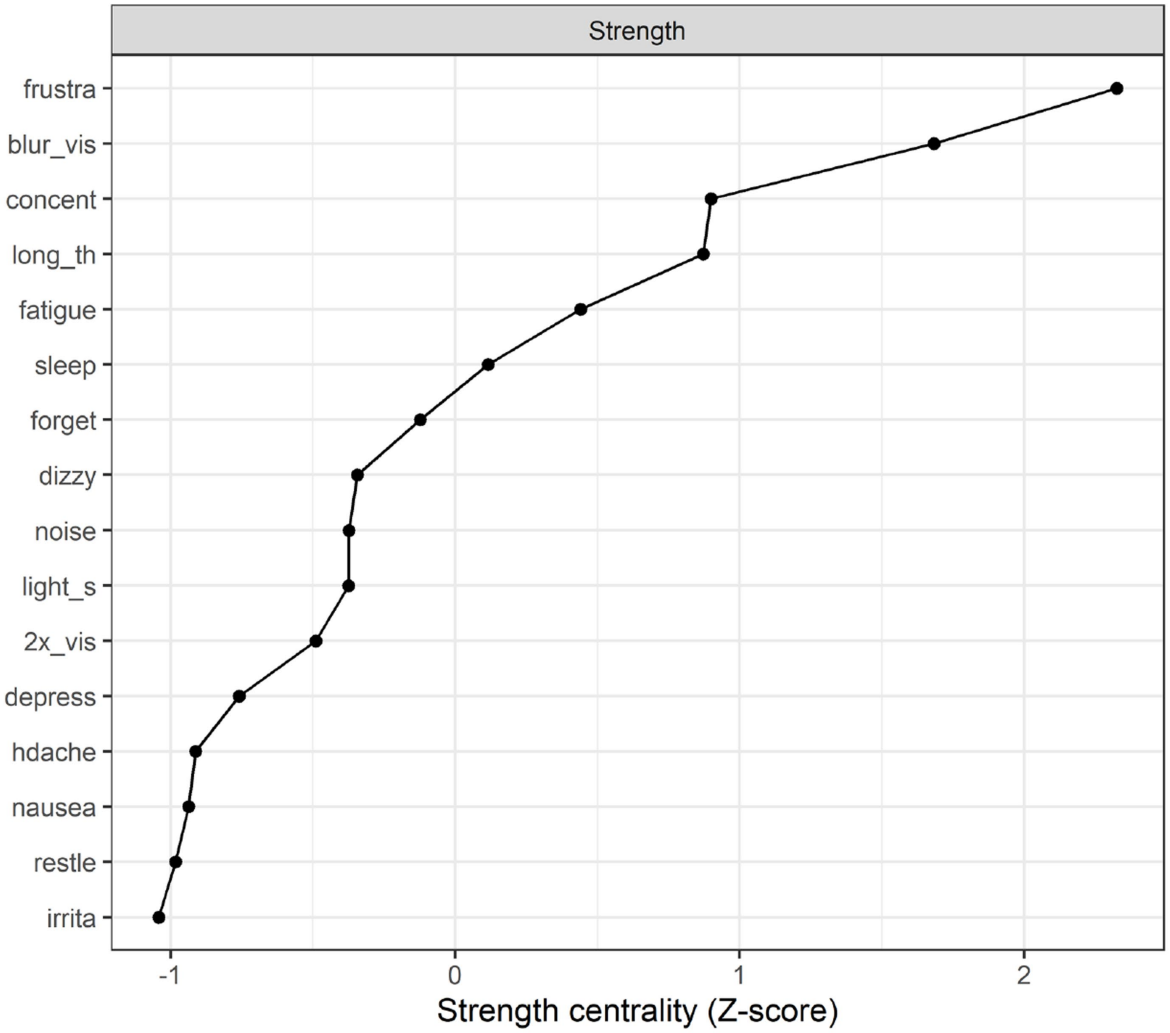
Strength centrality plot for the treatment seeking sample.

**Strength centrality plot**. A strength centrality plot was also generated (see Figure 2). The strength centrality of each node is the sum of its connections (i.e., partial correlations) to other nodes. These were then converted to *z*-scores for easier interpretation. The centrality plot for the treatment-seeking sample suggested that the most central symptom was frustration, followed by blurred vision, poor concentration, and taking longer to think. The least central symptom was irritability, although the centrality estimates were not drastically different across items.

**Network stability**. The stability analyses indicated that (across 1,000 simulations), randomly dropping up to 59.3% of cases resulted in edge weights that retained a correlation of at least 0.7 with those in the original sample. The same percentage (59.3%) could be dropped while retaining a correlation of 0.7 between the strength estimates in the original sample and those in the trimmed sample. This suggests relatively good stability (Epskamp et al. (34) suggest that these percentages should preferably be above 50%).

**Fit of network model**. The covariance matrix between items implied by the network model was a relatively strong fit to the sample covariance matrix. A root mean square error of approximation (RMSEA) of 0.00 indicated relatively essential no error relative to model complexity; an RMSEA of less than 0.05 would typically be considered to indicate good fit (50). The comparative fit index (CFI) of 1.00 indicated extremely good fit relative to an independence model (a CFI of greater than 0.95 would typically be considered to indicate good fit). The chi-square statistic of *χ*^2^(45) = 43.85, *p* = 0.52 indicated that a null hypothesis of perfect fit in the population could not be rejected. Importantly, these fit statistics are vulnerable to overfitting due to the fact that they were calculated using the same data used to select parameters and fit the model.

**Figure 3:**
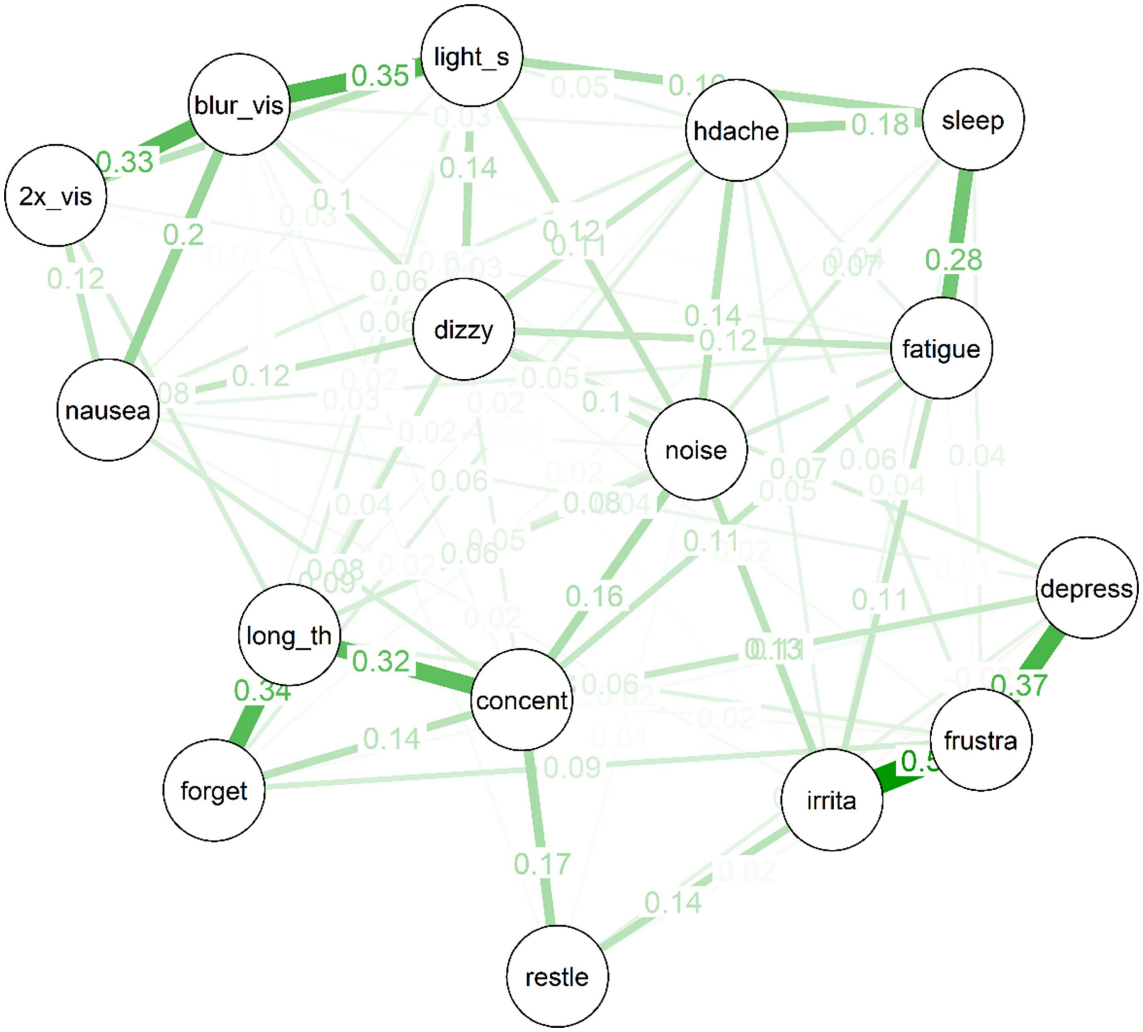
Partial correlation network for 10 year post-mTBI sample, the width, and saturation of edges are proportional to their strength.

### 10 years post mTBI

The edges for the network of post-concussive symptoms for the 10 year post mTBI sample were selected via the EBICglasso algorithm. Edges were then estimated using full information maximum likelihood.

Several interesting features are apparent in the network for the 10 year post-mTBI sample (See Figure 3). As was the case for the treatment-seeking sample, items cluster to some extent into similar “communities”: For example, the mood symptoms of depressed mood, frustration and irritability are strongly connected. So too are the cognitive symptoms of forgetfulness, poor concentration, and “taking longer to think.” There is also a cluster of physical symptoms (e.g., double vision, nausea, and light sensitivity) clustered around “blurred vision.” Second, there are no negative (red) partial correlations in the network. Third, the network is moderately sparse: Of a possible (16*15)/2 = 120 edges between items, the EBICglasso selected 77 edges, while the remaining 43 were constrained to zero, and are not displayed in the network plot.

**Figure 4:**
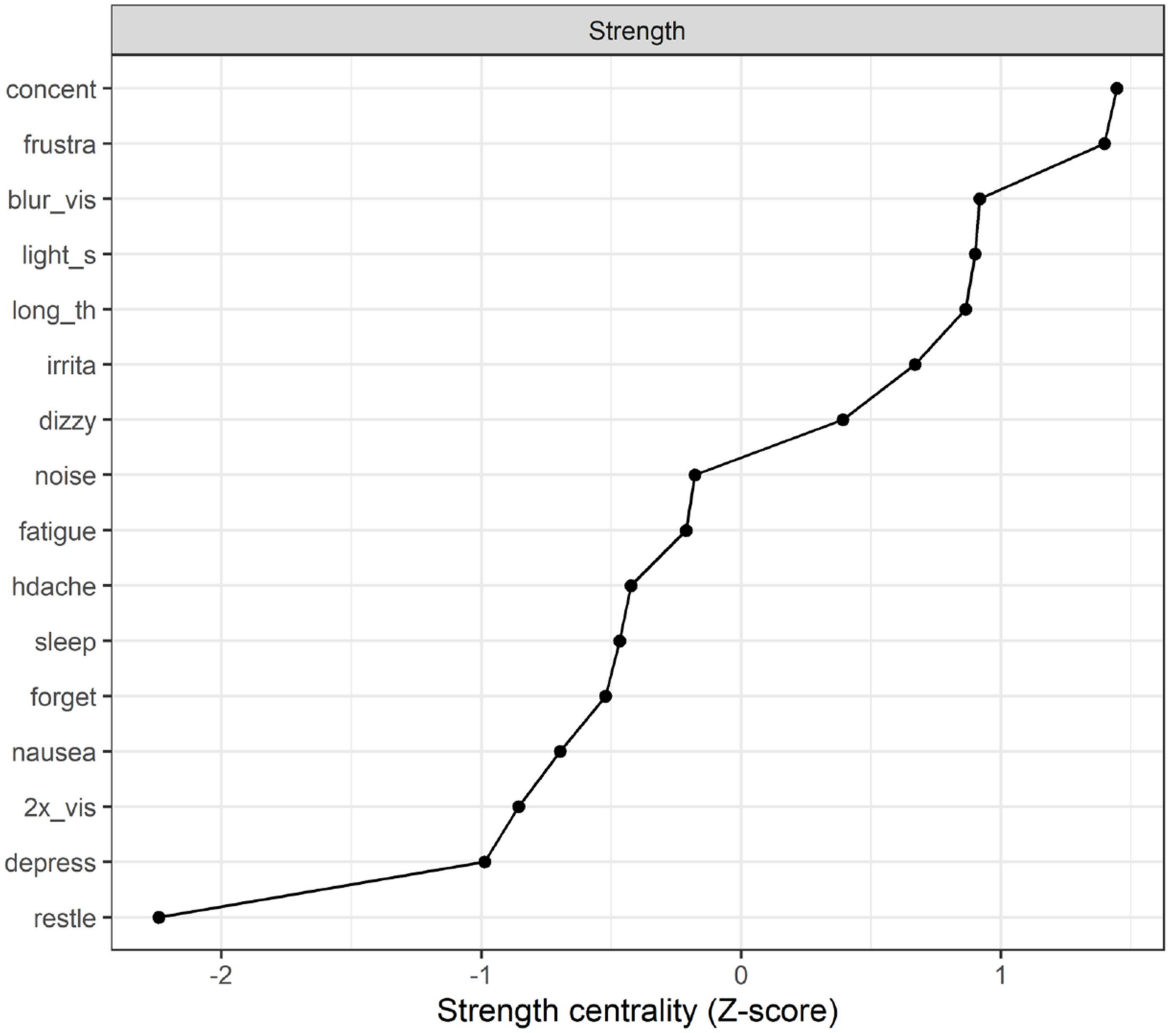
Strength centrality plot for the 10  year post mTBI group.

**Strength centrality plot**. The strength centrality plot (see Figure 4) suggested that the three most central symptoms for the 10 year post mTBI sample were poor concentration. Frustration and blurred vision. These nodes were also the most central in Study 1 (albeit in a different order). The least central symptom (by some margin) was restlessness (the strength centrality of each node is the sum of its connections (i.e., partial correlations) to other nodes. These were then converted to *z*-scores for easier interpretation).

**Network stability**. Stability analyses indicated that (across 1,000 simulations), randomly dropping up to 35.9% of cases resulted in edge weights and strength centrality estimates that each retained a correlation of at least 0.7 with those in the original sample. This suggests relatively moderate stability, a phenomenon presumably in large part to the constrained sample size.

**Fit of network model**. The covariance matrix between items implied by the network model was a relatively strong fit to the sample covariance matrix. The RMSEA of 0.016 indicated relatively low error relative to model complexity, while the comparative fit index (CFI) of 1.0 indicated extremely good fit relative to an independence model. The chi-square statistic of *χ*^2^(43) = 45.06, *p* = 0.39 indicated that a null hypothesis of perfect fit in the population could not be rejected. These fit statistics are nevertheless vulnerable to overfitting due to the fact that they were calculated using the same data used to select parameters and fit the model.

### Network comparison

The Network Comparison Test package in R (51) permits comparing network structure, edge strength, and global strength across networks. In a brief exploratory analysis we, therefore, compared the networks of the treatment-seeking and 10 year post mTBI samples. We found evidence of a difference in network structure across the two samples (*p* = 0.045). We found no evidence that the global strength (i.e., sum of absolute values of all edges) was different across groups: Sum treatment seeking = 7.08, sum 10 year post mTBI = 7.28, *S* = 0.197, *p* = 0.524. The only individual symptoms for which there was a significant difference in strength centrality between the two groups was irritability (*p* = 0.046, being higher in the 10 year post mTBI) and restlessness (*p* = 0.033, being higher in the 10 year post mTBI). For just a small minority of specific edges (16), there was a significant difference in edge weight between the treatment-seeking and 10 year post mTBI samples.

## Discussion

The overall objective of the current study was to apply network analysis to post-concussion symptoms after mTBI. To achieve this, two civilian samples were used: individuals seeking treatment for mTBI who were on average 6 weeks post-injury, and individuals who had experienced mTBI 10 years prior. The latter sample provides a unique opportunity to examine the course of post-concussion symptoms in the longer term, and also explore possible differences in post-concussion symptom networks over time. Network analyses in both samples had good network fit and adequate network stability indicating the suitability of applying this approach to post-concussion symptoms as measured with the RPQ. There have been recent calls to use network analysis with post-concussion symptoms because these symptoms are not exclusively driven by a latent disease entity, but are also strongly interrelated, activating, amplifying, and mutually reinforcing (31, 37, 38). Our findings support the application of this approach to post-concussion symptoms after mTBI.

Network analysis identifies symptoms that are central to the network. A symptom can display high centrality due to being a common cause of many other symptoms or being affected *by* many other symptoms. Symptom centrality provides a preliminary basis for hypothesis-generation regarding symptoms which may be fruitful targets for clinical intervention given the widespread effect they have on the entire network of symptoms. In the treatment-seeking sample, the most central symptoms were frustration, followed by blurred vision, poor concentration, and taking longer to think. Interestingly, when considering the network of post-concussion symptoms in the 10 year post-injury sample the symptoms central to the network were consistent with the treatment-seeking sample, although the order of centrality differed. In this cohort poor concentration was most central followed by frustration and blurred vision. The possible role of each of these symptoms on the network of post-concussion symptoms will now be discussed.

First, in regards to frustration, there is a wealth of evidence that acute psychological distress contributes to the development and maintenance of post-concussion symptoms (3, 28, 29, 52). This distress may be precipitated by the pathophysiology of mTBI influencing emotion centres within the cerebral cortex (i.e., limbic system, prefrontal cortex), as well as challenges adjusting to the ongoing effects of the injury and its implications on functioning (53–55). This distress may also be higher in those with a pre-injury mental health diagnosis, a robust prognostic risk factor for poorer recovery after mTBI (3, 13, 56). Our results demonstrate the relationship between frustration and many other post-concussions symptoms; that is, when frustration is heightened other post-concussion symptoms are also likely to be experienced as more severe. In addition, the central role of frustration on the network of post-concussion symptoms was still evident in the 10 year post-injury sample which suggests that these relationships may persist over time. As our study adopts a cross-sectional design, it is not possible to infer the directionality of the relationship between frustration and post-concussion symptoms within the network. However, it could be speculated that a bidirectional relationship exists where frustration influences symptoms, i.e., concentration difficulties and the presence of post-concussion symptoms increases the intensity of frustration, which is then reinforced over time.

The finding of blurred vision as a central symptom within the treatment-seeking sample and 10 year post-injury sample is intriguing. The visual system has expansive anatomy and physiology throughout the brain (57). The large vision network requires efficient neural interconnections and processing from multiple areas of the brain including frontal and posterior cerebral cortices, cranial nerves, and axonal interconnections (58). It is therefore not surprising that the visual system may be particularly vulnerable to the effects of brain injury (59). In regards to mTBI, vision system impairments have been identified in the first 2 weeks following injury (60) as well as in those with prolonged recovery (e.g., up to 12 months) (61, 62). Our findings provide initial evidence of the possible consequential impact of visual disturbances, more specifically blurred vision, on other post-concussion symptoms possibly contributing to their severity and persistence over time. Again, the directionality of these relationships cannot be confidently inferred in this study, but there is evidence in the literature to suggest that these associations could be bidirectional. Studies have found that blurred or double vision contributes to fatigue, concentration and reading difficulties (63, 64), whereas attentional and executive dysfunction can cause visual disturbances (65). Network analysis illustrates the role these symptoms have within the entire network of post-concussion symptoms. Our findings in conjunction with the existing literature thus suggest that from a rehabilitation perspective, when individuals are seeking treatment for mTBI there may be benefit in targeting blurred vision given the widespread effect this symptom may have on the entire network of symptoms. Currently, findings pertaining to the efficacy of treatments of visual disturbances is mixed (66, 67). However, these findings support calls for ongoing research specifically into visual rehabilitation in mTBI given the possible influential effect that difficulties in this area have on the constellation of post-concussion symptoms.

Finally, concentration difficulties also emerged as a central symptom in the network analysis and was the most central symptom in the 10 year post-injury sample. The significant role of cognitive difficulties on mTBI long-term health outcomes was highlighted in a systematic review of prognostic models of mTBI. Silverberg et al. (3) found that early-post injury neuropsychological functioning was one of the most robust prognostic factors of mTBI outcomes. Although “neuropsychological functioning” was not defined, this may well comprise concentration difficulties given the fundamental role that this cognitive process has on wider cognitive functioning. These sequalae may be associated with the acute pathophysiology of mTBI (68, 69). In addition, concentration difficulty is a post-concussion symptom highly susceptible to the impact of other difficulties such as pain, fatigue, neurological symptoms, as well as mental distress (70). This may account for its central role within the post-concussion symptoms network.

In summary, the findings of the current study suggest that poor concentration, frustration and blurred vision may have an influential role on post-concussion symptoms, as well as their presence over time given their centrality within the network of symptoms. Not only were these symptoms highly central when individuals are approximately 6 weeks post-injury, but also even when individuals are 10 years post-injury. This finding provides initial unique insights to aid our understanding of how post-concussion symptoms develops and how they may be maintained over time. From a rehabilitation perspective, if future evidence confirms that frustration, concentration difficulties and blurred vision are central due to having causal effects *on* other symptoms, prioritising the treatment of these three symptoms may trigger the greatest reduction in overall post-concussion symptoms. Finally, the interplay between these symptoms over time may offer valuable insights into the distinct recovery pathways experienced after mTBI and the factors influencing participation in rehabilitation.

Currently, the application of network analysis to post-concussion symptoms has produced mixed and inconsistent findings. In accordance with our results, Goodwin et al. (38) found that difficulties concentrating were the most central symptom in high school athletes with suspected sports-related concussion. Additionally, Iverson et al. (35) found that “feeling more emotional” was a central baseline symptom in adolescents with ADHD; however, dizziness was also central which was also found in Preszler et al. (37) network analysis of adolescents recruited from a concussion speciality clinic. It is difficult to make direct inferences between our findings and the current evidence given the relatively narrow populations used. Differences in the centrality of symptoms may be indicative of the unique characteristics of the sample being analysed. The use of network analysis in post-concussion symptoms is in its infancy and future research is needed to examine this approach. It may be the case that a “one size fits all” network is not evident across the spectrum of mTBI, and differing networks may be revealed across different cohorts of individuals who have experienced a mTBI. For example, a post-concussion symptoms network for adolescents with mTBI may be different from a network of post-concussion symptoms for adults. One limitation of network analysis, is that its flexibility and complexity may mean that estimated networks are vulnerable to poor replicability (46). One strength of this paper is that we have used two different samples of individuals who have experienced a mTBI and yet the centrality in symptoms was very consistent. Although further replication is required, it does provide initial support for the findings of the role of these symptoms in the development and maintenance of post-concussion symptoms over time.

It is interesting when comparing the overall network of symptoms between the treatment-seeking sample to those who experienced their mTBI 10 years earlier, the centrality of symptoms remained fairly consistent with significant differences found only in irritability and restlessness. This finding suggests that the relationship between post-concussion symptoms may remain, for the most part, somewhat constant over time. However, it is currently not possible to infer the direct role that mTBI has had on the network of symptoms or these relationships. An ongoing challenge faced in our understanding of post-concussion symptoms is that all symptoms are non-specific and it is therefore difficult to tease out the causal effects of injury vs. non-injury factors (18). This highlights an important limitation that must be considered when interpreting the results of this study. Several non-symptom variables were absent from the network that could influence the relationship between symptoms, e.g., age, gender, concussion and physical health history. More specifically, it may be the case that certain individuals, have pre-existing ‘vulnerable’ networks where the strength of the relationship between certain symptoms are already evident and thus continue to persist over time. It may also be that the relationship between symptoms, or the centrality of symptoms changes as a consequence of a mTBI and these changes persist overtime. These changes may also be maintained by other non-injury-related factors (14). One of these factors that is particularly worthy of highlighting is presence of a pre-injury psychiatric diagnosis given the impact this could have on the network of symptoms. Pre-injury mental health conditions are robust predictors of mTBI outcomes, affecting incidence (71), severity (72), and duration of symptoms (73, 74). In support of the potential confounding role this factor may have on the interrelationship between symptoms, Fonda et al. (75) recently applied network analysis to post-concussion symptoms and comorbid psychiatric conditions in veterans and service members. Exploratory factor analysis was used to identify and define nodes to include in the network analysis based on a range of self-report measures. In this study, mTBI military count was included in the network analysis and was found to be the least influential node in the network. The authors also compared networks between those that had sustained a mTBI (*n* = 322) and those that had not (*n* = 297), and found that comparable network structures were evident between the two groups. It was consequently concluded that the pattern of symptoms prevalent in the sample may be largely independent of mTBI and psychiatric conditions may be the most influential factor in their development and maintenance. Thus, inclusion of potential confounding variables, such as pre-injury psychiatric conditions in future research using network analysis is essential to ensure that an understanding of all the factors, not just mTBI, that influence the network of post-concussion symptoms is understood.

The current study is also limited in that a single self-report tool was used to ascertain post-concussion symptom severity and thus post-concussion symptom severity was ascertained by using a single item that assesses symptom severity in the past 24 h. Bias in symptom reporting, as well as constraints imposed by a single self-report measure, could impact the validity of the data. Recently, Preszler et al. (37) included clinical assessments of vestibular and oculomotor functioning in their network analysis of post-concussion symptoms. In addition, the time constraint of the measure used could impact the accurate attainment of overall symptom experience. Future research would benefit from including data on post-concussion symptoms gathered from multiple methodologies, i.e., cognitive test scores or structured interviews of psychological disorders, as well as over an extended period of time. Specific information pertaining to the injury characteristics of these samples (i.e., Glasgow Coma Scale (GCS), duration of post-traumatic amnesia (PTA) or loss of consciousness (LOC), presence of intracranial injury) was not available. Given the implications these could have on symptom manifestation and severity, future research should include these variables when conducting network analyses. The dataset for both samples was only moderate in size, although the bootstrap analyses supported the stability of the results despite the sample sizes. Future studies could benefit from applying the Monte Carlo method for sample size determination for network analyses recently developed by Constantin et al. (76). Finally, it is important to bear in mind the characteristics of the samples and the implications this could have on the generalisability of the findings. The treatment-seeking sample had a higher rate of females with the most common cause of injury being either hit by an object or a fall. This is not consistent with the characteristics of mTBI more generally (42) and may therefore represent a unique cohort who are needing treatment. In lieu of this, the study findings should be treated cautiously and future research which addresses these limitations, is needed to ensure replicability of these results.

In conclusion, network analysis applied to post-concussion symptoms is a promising approach and may provide novel insights into our understanding of post-concussion symptoms and potentially aid in prioritisation and rehabilitation of these symptoms. We found that in a mTBI treatment seeking sample frustration, blurred vision and concentration difficulties were the most central symptoms within the network. These symptoms were also most central in those who were 10 years post mTBI. We also found few significant differences when the two networks were compared, suggesting that the relationship between symptoms within this network may remain somewhat constant over time. However, the use of network analysis in post-concussion symptoms is currently limited and future research is needed to replicate the current findings, compare networks with different cohorts of individuals who have experienced mTBI, as well as examine the direct role that mTBI has within the network.

## Data availability statement

The raw data supporting the conclusions of this article will be made available by the authors, without undue reservation.

## Ethics statement

Ethical approval for the treatment seeking sample were received from New Zealand’s National Health and Disability Ethics Committee (ref 18/CEN/79) and the Auckland University of Technology Ethics Committee (ref 20/32). For the 10 year post-mTBI sample ethical approval was received Health and Disability Ethics Committee (ref 19NTB166) and Auckland University of Technology Ethics Committee AUTEC 9 (ref 19/408). The patients/participants provided their written informed consent to participate in this study.

## Author contributions

JF, DS, AT, and MW drafted and designed the study. MW designed and executed the statistical analyses. JF, DS, and AT collected data for the study. JF wrote an initial manuscript draft. DS, AT, and MW revised and approved the final manuscript. All authors contributed to the article and approved the submitted version.

## Funding

This research was supported by grants from the Health Research Council of NZ (treatment-seeking sample: ref. 18/046; 20/041) and a Rutherford discovery fellowship administered by the Royal Society Tē Apārangi (10 year post-injury sample).

## Conflict of interest

The authors declare that the research was conducted in the absence of any commercial or financial relationships that could be construed as a potential conflict of interest.

## Publisher’s note

All claims expressed in this article are solely those of the authors and do not necessarily represent those of their affiliated organizations, or those of the publisher, the editors and the reviewers. Any product that may be evaluated in this article, or claim that may be made by its manufacturer, is not guaranteed or endorsed by the publisher.
