# Supplementary material for: Network analysis applied to post-concussion symptoms in two mild traumatic brain injury samples
Source: Front Neurol. 2023 Jul 20;14:1226367. doi: 10.3389/fneur.2023.1226367 (PMC10398392; doi:10.3389/fneur.2023.1226367)

Supplementary Materials

This Supplementary Materials document includes additional statistical output that was not included in the main text for reasons of brevity.

# Correlation Matrices

Zero-order correlation matrices for responses to the 16 items in the Rivermead Post Concussion Symptom Scale can be found in Supplementary Table 1 (treatment-seeking sample) and Supplementary Table 2 (10-year sample). These correlation matrices indicates that responses to all pairs of items were positively correlated, with many of these correlations being quite substantial.

Supplementary Table 1
*Correlation Matrix for Rivermead Items – (Treatment-Seeking Sample)*

| No. | Item | 1 | 2 | 3 | 4 | 5 | 6 | 7 | 8 | 9 | 10 | 11 | 12 | 13 | 14 | 15 | 16 |
| --- | --- | --- | --- | --- | --- | --- | --- | --- | --- | --- | --- | --- | --- | --- | --- | --- | --- |
| 1 | Headaches | 1.00 | 0.35 | 0.36 | 0.51 | 0.46 | 0.51 | 0.35 | 0.30 | 0.39 | 0.42 | 0.46 | 0.48 | 0.35 | 0.44 | 0.22 | 0.36 |
| 2 | Dizziness | 0.35 | 1.00 | 0.54 | 0.40 | 0.34 | 0.33 | 0.31 | 0.35 | 0.35 | 0.45 | 0.44 | 0.42 | 0.47 | 0.34 | 0.34 | 0.35 |
| 3 | Nausea | 0.36 | 0.54 | 1.00 | 0.35 | 0.30 | 0.33 | 0.22 | 0.32 | 0.26 | 0.36 | 0.34 | 0.37 | 0.35 | 0.40 | 0.33 | 0.32 |
| 4 | Noise | 0.51 | 0.40 | 0.35 | 1.00 | 0.41 | 0.48 | 0.38 | 0.29 | 0.42 | 0.45 | 0.47 | 0.51 | 0.24 | 0.57 | 0.13 | 0.37 |
| 5 | Sleep | 0.46 | 0.34 | 0.30 | 0.41 | 1.00 | 0.48 | 0.50 | 0.44 | 0.48 | 0.44 | 0.41 | 0.43 | 0.30 | 0.33 | 0.23 | 0.56 |
| 6 | Fatigue | 0.51 | 0.33 | 0.33 | 0.48 | 0.48 | 1.00 | 0.39 | 0.36 | 0.53 | 0.47 | 0.60 | 0.55 | 0.24 | 0.42 | 0.11 | 0.45 |
| 7 | Irritability | 0.35 | 0.31 | 0.22 | 0.38 | 0.50 | 0.39 | 1.00 | 0.50 | 0.62 | 0.45 | 0.44 | 0.44 | 0.20 | 0.28 | 0.16 | 0.31 |
| 8 | Mood | 0.30 | 0.35 | 0.32 | 0.29 | 0.44 | 0.36 | 0.50 | 1.00 | 0.61 | 0.42 | 0.39 | 0.41 | 0.20 | 0.25 | 0.26 | 0.33 |
| 9 | Frustration | 0.39 | 0.35 | 0.26 | 0.42 | 0.48 | 0.53 | 0.62 | 0.61 | 1.00 | 0.55 | 0.55 | 0.53 | 0.17 | 0.39 | 0.13 | 0.47 |
| 10 | Memory | 0.42 | 0.45 | 0.36 | 0.45 | 0.44 | 0.47 | 0.45 | 0.42 | 0.55 | 1.00 | 0.70 | 0.70 | 0.40 | 0.40 | 0.28 | 0.41 |
| 11 | Concentration | 0.46 | 0.44 | 0.34 | 0.47 | 0.41 | 0.60 | 0.44 | 0.39 | 0.55 | 0.70 | 1.00 | 0.75 | 0.43 | 0.48 | 0.22 | 0.46 |
| 12 | Thinking_speed | 0.48 | 0.42 | 0.37 | 0.51 | 0.43 | 0.55 | 0.44 | 0.41 | 0.53 | 0.70 | 0.75 | 1.00 | 0.41 | 0.48 | 0.23 | 0.49 |
| 13 | Blurred_vision | 0.35 | 0.47 | 0.35 | 0.24 | 0.30 | 0.24 | 0.20 | 0.20 | 0.17 | 0.40 | 0.43 | 0.41 | 1.00 | 0.39 | 0.59 | 0.28 |
| 14 | Light | 0.44 | 0.34 | 0.40 | 0.57 | 0.33 | 0.42 | 0.28 | 0.25 | 0.39 | 0.40 | 0.48 | 0.48 | 0.39 | 1.00 | 0.20 | 0.45 |
| 15 | Double_vision | 0.22 | 0.34 | 0.33 | 0.13 | 0.23 | 0.11 | 0.16 | 0.26 | 0.13 | 0.28 | 0.22 | 0.23 | 0.59 | 0.20 | 1.00 | 0.20 |
| 16 | Restless | 0.36 | 0.35 | 0.32 | 0.37 | 0.56 | 0.45 | 0.31 | 0.33 | 0.47 | 0.41 | 0.46 | 0.49 | 0.28 | 0.45 | 0.20 | 1.00 |

Supplementary Table 2
*Correlation Matrix for Rivermead Items – (10 Years Post-Injury Sample)*

| No. | Item | 1 | 2 | 3 | 4 | 5 | 6 | 7 | 8 | 9 | 10 | 11 | 12 | 13 | 14 | 15 | 16 |
| --- | --- | --- | --- | --- | --- | --- | --- | --- | --- | --- | --- | --- | --- | --- | --- | --- | --- |
| 1 | Headaches | 1.00 | 0.43 | 0.34 | 0.44 | 0.44 | 0.39 | 0.38 | 0.28 | 0.39 | 0.37 | 0.34 | 0.39 | 0.37 | 0.42 | 0.27 | 0.17 |
| 2 | Dizziness | 0.43 | 1.00 | 0.43 | 0.45 | 0.30 | 0.44 | 0.37 | 0.34 | 0.39 | 0.43 | 0.46 | 0.46 | 0.49 | 0.52 | 0.38 | 0.18 |
| 3 | Nausea | 0.34 | 0.43 | 1.00 | 0.35 | 0.26 | 0.34 | 0.30 | 0.27 | 0.30 | 0.23 | 0.39 | 0.28 | 0.51 | 0.44 | 0.42 | 0.10 |
| 4 | Noise | 0.44 | 0.45 | 0.35 | 1.00 | 0.39 | 0.38 | 0.44 | 0.24 | 0.40 | 0.33 | 0.50 | 0.46 | 0.40 | 0.47 | 0.29 | 0.26 |
| 5 | Sleep | 0.44 | 0.30 | 0.26 | 0.39 | 1.00 | 0.50 | 0.37 | 0.26 | 0.36 | 0.32 | 0.36 | 0.32 | 0.32 | 0.44 | 0.21 | 0.18 |
| 6 | Fatigue | 0.39 | 0.44 | 0.34 | 0.38 | 0.50 | 1.00 | 0.42 | 0.28 | 0.39 | 0.36 | 0.45 | 0.44 | 0.35 | 0.38 | 0.31 | 0.10 |
| 7 | Irritability | 0.38 | 0.37 | 0.30 | 0.44 | 0.37 | 0.42 | 1.00 | 0.41 | 0.70 | 0.32 | 0.41 | 0.38 | 0.32 | 0.30 | 0.26 | 0.34 |
| 8 | Mood | 0.28 | 0.34 | 0.27 | 0.24 | 0.26 | 0.28 | 0.41 | 1.00 | 0.57 | 0.34 | 0.41 | 0.28 | 0.23 | 0.15 | 0.11 | 0.25 |
| 9 | Frustration | 0.39 | 0.39 | 0.30 | 0.40 | 0.36 | 0.39 | 0.70 | 0.57 | 1.00 | 0.42 | 0.45 | 0.45 | 0.30 | 0.31 | 0.25 | 0.31 |
| 10 | Memory | 0.37 | 0.43 | 0.23 | 0.33 | 0.32 | 0.36 | 0.32 | 0.34 | 0.42 | 1.00 | 0.53 | 0.61 | 0.34 | 0.38 | 0.26 | 0.10 |
| 11 | Concentration | 0.34 | 0.46 | 0.39 | 0.50 | 0.36 | 0.45 | 0.41 | 0.41 | 0.45 | 0.53 | 1.00 | 0.64 | 0.40 | 0.43 | 0.32 | 0.37 |
| 12 | Thinking_speed | 0.39 | 0.46 | 0.28 | 0.46 | 0.32 | 0.44 | 0.38 | 0.28 | 0.45 | 0.61 | 0.64 | 1.00 | 0.41 | 0.45 | 0.38 | 0.23 |
| 13 | Blurred_vision | 0.37 | 0.49 | 0.51 | 0.40 | 0.32 | 0.35 | 0.32 | 0.23 | 0.30 | 0.34 | 0.40 | 0.41 | 1.00 | 0.65 | 0.59 | 0.21 |
| 14 | Light | 0.42 | 0.52 | 0.44 | 0.47 | 0.44 | 0.38 | 0.30 | 0.15 | 0.31 | 0.38 | 0.43 | 0.45 | 0.65 | 1.00 | 0.51 | 0.13 |
| 15 | Double_vision | 0.27 | 0.38 | 0.42 | 0.29 | 0.21 | 0.31 | 0.26 | 0.11 | 0.25 | 0.26 | 0.32 | 0.38 | 0.59 | 0.51 | 1.00 | 0.11 |
| 16 | Restless | 0.17 | 0.18 | 0.10 | 0.26 | 0.18 | 0.10 | 0.34 | 0.25 | 0.31 | 0.10 | 0.37 | 0.23 | 0.21 | 0.13 | 0.11 | 1.00 |

# Treatment-Seeking Sample – Network Analysis with DWLS

Parameters for the network analysis reported in the main text were estimated via full information maximum likelihood. This permitted the presence of missing data but has the limitation of assuming the variables have a multivariate normal distribution. Taken literally, this assumption is breached because the original data stems from ordered-categorical items (whereas the multivariate normal distribution is continuous). We also completed an alternative version of our analyses using diagonally weighted least squares (DWLS) estimation, which does not permit missing data, but does not assume multivariate normality. This model still relies on the parameter selection via EBICglasso (as reported in the main manuscript) so this produces the same edges, but different edge weights and other derived statistics. Using this analysis resulted in the sample size decreasing from 258 to 242 due to 16 participants have some missing responses.

The partial correlation network resulting from this analysis is presented in Supplementary Figure 1. As was the case in the main analysis (using FIML), items cluster to some extent into “communities”: For example, the mood symptoms of depressed mood, frustration and irritability are clearly connected. So too are the cognitive symptoms of forgetfulness, poor concentration, and “taking longer to think.” Again, almost all of the edges are positive, with the exception of a single negative edge between blurred vison and frustration.

A strength centrality plot is presented in Supplementary Figure 2. Frustration remained the most central symptom, although there were some slight changes to the rank-order of some of the other symptoms. For example, blurred vision dropped from the second-most to third-most central symptom. Overall, the results of this robustness analysis using DWLS estimation produced very similar—but not identical—findings.

Supplementary Figure 1
*Treatment-Seeking Sample Partial Correlation Network (Estimated via DWLS)*


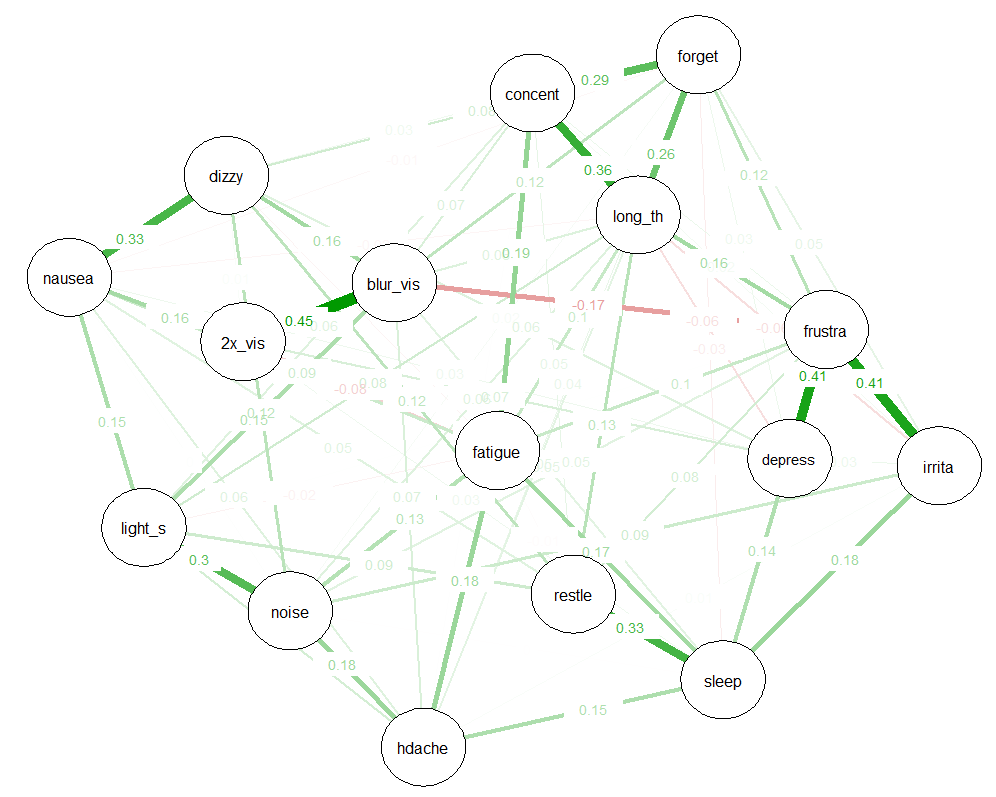


The covariance matrix between items implied by the network model was a relatively strong fit to the sample covariance matrix. The RMSEA of 0.00 indicated very low error relative to model complexity, while the comparative fit index (CFI) of 1.00 indicated extremely good fit relative to an independence model. The chi-square statistic of χ^2^(45) = 9.70, *p* = 1.00 indicated that a null hypothesis of perfect fit in the population could not be rejected.

Supplementary Figure 2
*Strength Centrality Plot for Treatment-Seeking Sample Network (Estimated via DWLS)*


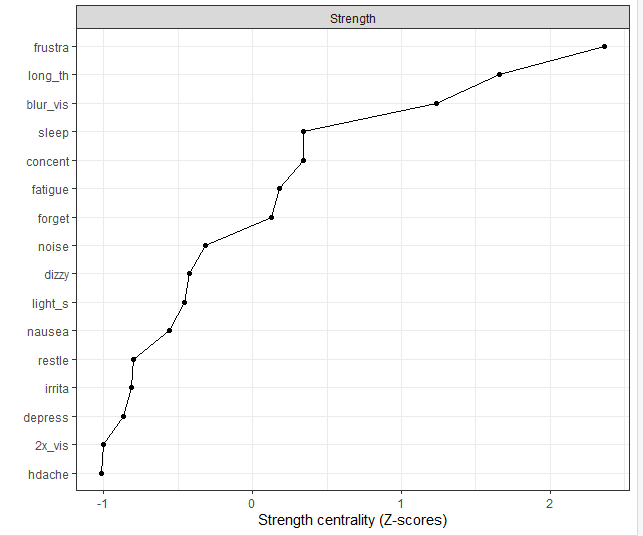


# 10 Year Sample – Network Analysis with DWLS

We likewise conducted a robustness analysis for the 10 year post-injury sample, where we estimated the same network using DWLS estimation. This resulted in the sample size dropping from *N* = 193 to *N* = 161 due to missing data. The resulting network is presented in Supplementary Figure 3. Similar communities of items appear as in the main analysis: For example, there is a clear community of the cognitive items forgetfulness, concentration difficulties, and taking longer to think.

Supplementary Figure 3
*10 Year Post-Injury Partial Correlation Network (Estimated via DWLS)*


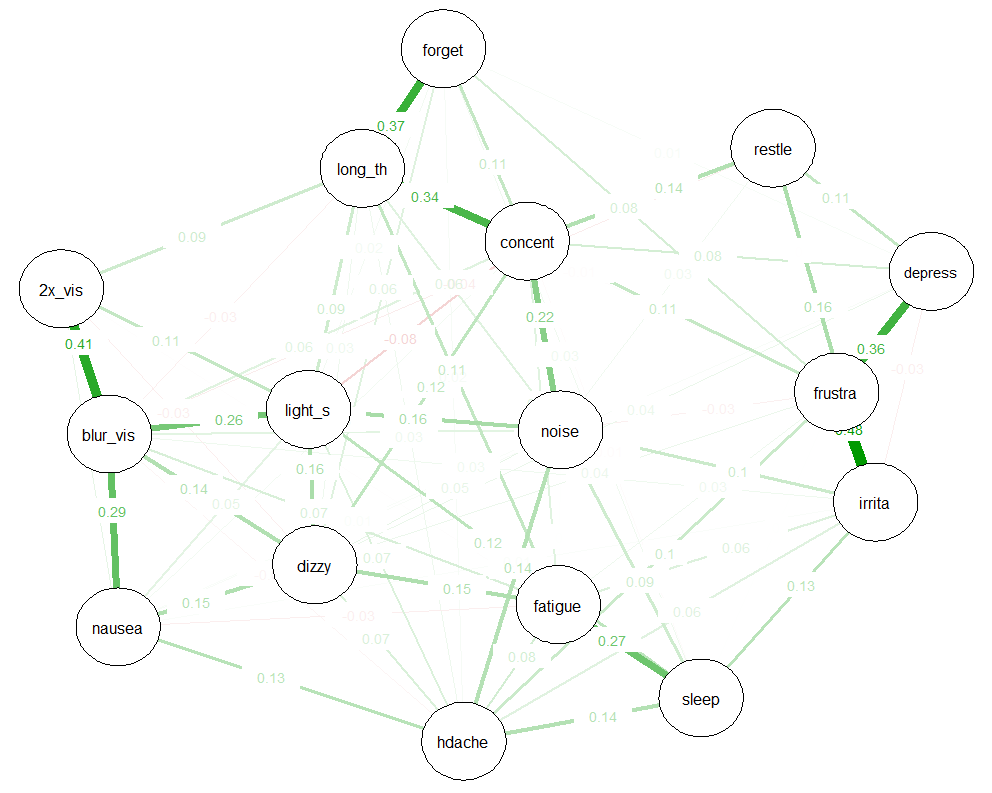


A strength centrality plot is displayed in Supplementary Figure 4. This again produces fairly similar results to the main analysis: For example, concentration difficulties, frustration and blurred vision remain the top three most central symptoms, albeit in a different order, and restlessness remains the least central.

The covariance matrix between items implied by the network model was a relatively strong fit to the sample covariance matrix. The RMSEA of 0.00 indicated very low error relative to model complexity, while the comparative fit index (CFI) of 1.00 indicated extremely good fit relative to an independence model. The chi-square statistic of χ^2^(43) = 15.26, *p* = 1.00 indicated that a null hypothesis of perfect fit in the population could not be rejected. Overall the findings remained substantively similar in this alternative robustness analysis.

Supplementary Figure 4
*Strength Centrality Plot for 10 Year Post-Injury Network (Estimated via DWLS)*


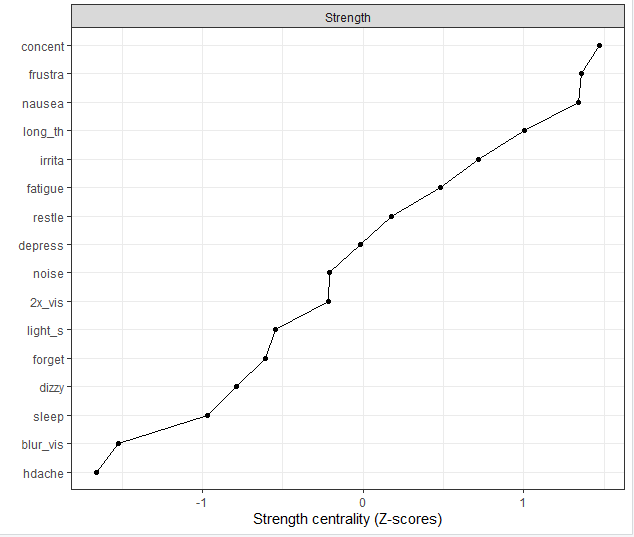

Supplement: Supplementary file 1 [file Table_1.DOCX]
